# Miscellaneous

**Published:** 1889-02

**Authors:** 


### MISCELLANEOUS.

If anyone is unfortunate enough to swallow poison of any kind through mistake or otherwise, give immediately two gills of sweet oil. It is an effectual antidote to almost any poison. Anyone with a strong constitution should take twice the quantity. (In liquid measure one teacupful is one gill.)

A curiously unpleasant peasant superstition has just been revealed at a trial in Southern Russia, which ended in the conviction of four peasants for the murder of a girl eleven years old. The superstition recalls that about thieves' candles narrated in connection with the Whitechapel murders. These peasants, it seems, were believers in the superstition that candles made of human fat rendered the bearers invisible. To obtain these articles they first attempted to murder a boy in a forest. They next tried to kill an old peasant, thirdly a Russian clergyman, and being disturbed on all three occasions, they at last succeeded in murdering Sukena Cherkaschina. With the fat from the child's body they made candles and with their help attempted to commit a robbery. The light of the candles betrayed their doings, and on being arrested they confessed everything. The evidence in court showed the belief in the thieves' candle superstition to be very widespread in Russia.

Nose Bleed.—A well-known physician states that while travelling in the north of Scotland last year, and being in the company of Sir Morell Mackenzie, then plain Dr. Mackenzie, late attendant of Kaiser Friedrich, he was attacked with a nose bleed and was obliged to retire from the room. He was shortly afterwards followed into the adjoining apartment by the medical knight, who told him of his simple plan of stopping the flow of blood. It is as follows : Grasp firmly the nose with the finger and thumb of the right hand for fully ten or fifeen minutes, thus completely stopping the movement of air through the nose. This will stop the hemorrhage without any danger of a renewed attack.

An Indian Boy's Composition : Story of Good Bird and Bad Cat.—One day, bright day, and a little bird happy and stood on a log, and sang all day long. That bird does not know anything about cat. She thinks nobody is near to her. But behind the near log one sly old cat is watching. She wants to eat for supper, and she thinks about stealing all the time. The old cat came very slow, and by-and-by she go after the little bird, but she does not see him, and sang aloud again. She sang just like this, "I am always try do what is right ; when I ever die I go to heaven," that bird said these all words, and I shall not forget the bird what it said, and these all words it said, and after two or three minutes go died ; that cat jumped and catch and kill—eat all up except left little things from bird, wings, legs or skin, and that bird is glad to die because she is very good bird. The little bird has last time sang, and very happy was the little bird after that. I think the old cat have good dinner and happy just same as the bird was at first time.—[This composition was written by an Indian boy at the Hampton School, and is published just as he wrote it. The optimism of it is delicious.—Eds. *Transcript.*]

Six Exquisite Water Colors.—It is strange that a Boston stove concern should issue the daintiest Calendar of the season, but so it is. The Smith & Anthony

Stove Co., of Boston, manufacturers of the celebrated Hub Ranges, have achieved this distinction. Their Calendar is in six sheets, tied together by a ribbon, each sheet being a fac-simile of a delicate water-color drawing, by Miss L. B. Humphrey. The designs are six charming sketches of child life, together with delicate landscape, exquisite in color and treatment. The complete set can be had by sending 25 cents in stamps or currency to the above address. As a work of art merely, it is desirable.

THE distressing diseases of children, such as whooping cough, etc., are of such daily occurrence, and the effects so often fatal, that any radical remedy for their relief and cure should be heartily welcomed by every parent in the land. The Enterprise Vapor Medicator, an explanation of which is to be found among our advertisements, is highly recommended by those who have used it.

ROUND SHOULDERS.—A stooping figure and a halting gait, accompanied by the unavoidable weakness of lungs incidental to a narrow chest, may be entirely cured by the very simple and easily performed exercise of raising one's self upon the toes leisurely in a perpendicular several times daily. To take this exercise properly one must take a perfect position, with the heels together and the toes at an angle of 45 degrees. Then drop the arms lifelessly by the sides, animating and raising the chest to its full capacity muscularly, the chin well drawn in and the crown of the head feeling, as our professor used to put it, as if attached to a string suspended from the ceiling above. Slowly raise upon the balls of both feet to the greatest possible height, thereby exercising all the muscles of the legs and body ; come again into the standing position, without swaying the body backward out of the perfect line. Repeat this same exercise, first on one foot, then on the other. It is wonderful what a straightening-out power this exercise has upon round shoulders and crooked backs, and one will be surprised to note how soon the lungs begin to show the effect of such expansive development.

